# Molecular Characterization of ‘*Candidatus* Phytoplasma prunorum’ in the Czech Republic and Susceptibility of Apricot Rootstocks to the Two Most Abundant Haplotypes

**DOI:** 10.3390/microorganisms12020399

**Published:** 2024-02-17

**Authors:** Tomáš Kiss, Dana Šafářová, Milan Navrátil, Tomáš Nečas

**Affiliations:** 1 Department of Fruit Science, Faculty of Horticulture, Mendel University in Brno, Zemědělská 1, 613 00 Brno, Czech Republic; tomas.necas@mendelu.cz; 2 Department of Cell Biology and Genetics, Faculty of Science, Palacký University in Olomouc, Šlechtitelů 27, 779 00 Olomouc, Czech Republic; dana.safarova@upol.cz (D.Š.); milan.navratil@upol.cz (M.N.)

**Keywords:** European stone fruit yellows disease, *Prunus*, phytoplasma quantification, genotyping, symptomatology

## Abstract

‘*Candidatus* Phytoplasma prunorum’ is one of the most destructive pathogens of *Prunus* species, where susceptible species render unproductive several years after infection. In epidemiology, the molecular characterization of phytoplasmas is based on sequence analysis of variable nonribosomal genes. In this study *aceF*, *pnp*, *imp* and *secY* genes were used for characterization of the ‘*Ca.* P. prunorum’ genotypes present in the Czech Republic. In total, 56 plant and 33 vector (*Cacopsylla pruni*) samples positive to ‘*Ca.* P. prunorum’ collected in seven localities were used in the study. Based on sequence analysis, four *aceF*, two *pnp*, six *imp*, and three *secY* genotypes were identified in analyzed samples. The most abundant in both plant and insect samples were the A6, P2, I4, and S2 genotypes. Most of the Czech ‘*Ca.* P. prunorum’ haplotypes clustered together in the haplotype network analysis. Next, two isolates representing the two most abundant Czech haplotypes (A6-P2-I4-S2 and A5-P2-I4-S2) were used in the susceptibility test of three apricot rootstock types (St. Julien A, M-VA-1, GF-305). Susceptibility was analyzed by phytoplasma quantification using quantitative real-time PCR and evaluation of symptom manifestation. Based on the results, the influence of the rootstock type on the phytoplasma titer and symptom manifestation was greater than of the phytoplasma isolate, while the year of analysis had no influence on the results. The results also showed that the phytoplasma titer is increasing in plant tissues during the vegetation period.

## 1. Introduction

‘*Candidatus* Phytoplasma prunorum’, the causal agent of European stone fruit yellows disease (ESFY), is one of the most destructive pathogens infecting fruit trees in the Europe and Mediterranean area [1]. It is a cell wall-less bacterium invading phloem tissues of *Prunus* species [2] and is naturally vectored from plant to plant by a psyllid *Cacopsylla pruni* (Scopoli, 1763) [3]. It belongs to the 16SrX (apple proliferation) group together with other important fruit tree phytoplasmas, ‘*Candidatus* Phytoplasma mali’, the causal agent of apple proliferation disease, and ‘*Candidatus* Phytoplasma pyri’, the causal agent of pear decline disease. The most common ESFY symptoms are leafroll and leaf yellowing or reddening during the growing season and leaf bud break before flowering during the dormant season. Infected trees have reduced yield and vitality [4], and the susceptible genotypes may become unproductive 8 to 10 years after planting [5].

Classification of phytoplasmas is based on analysis of 16S rRNA or conserved/housekeeping genes, phytopathological and genomic differences [2,6]. In epidemiological studies, the analysis of the 16S rRNA gene is not sufficient for strain differentiation [7] and, therefore, other genes have been investigated. In the last two decades, a considerable effort has been made to identify less or more conservative genes suitable for detailed identification of ‘*Ca*. P. prunorum’ strains. Ribosomal genes such as *rpsC* [8], but mainly non-ribosomal genes such as *aceF*, *pnp*, *imp*, *secY*, [9], *tuf*, *tylC* [10], *fol* [8] and *hflb* [11] were studied. Among these, the highest variability was observed in the sequences of the *aceF* and *imp* genes where 12 and 25 genotypes, respectively, were identified up to date [9,12,13]. The *acef* gene encodes the dihydrolipoamide acetyltransferase component of pyruvate dehydrogenase involved in sugar metabolism [14]. The *imp* gene encodes an immunodominant membrane protein located in the outer part of the plasma membrane involved in the phytoplasma and host cell interaction [15]. The next most studied but less variable genes are *secY* encoding a transmembrane subunit of the secretion system [16] and *pnp* encoding polynucleotide phosphorylase [17], where three and two genotypes have been identified, respectively [9]. Sequence analysis of the *aceF* and *imp* genes has been used in studies focused on the geographical distribution of ‘*Ca*. P. prunorum’ genotypes but also on the identification of strains with different virulence [9,11,12,13,18]. Together with sequences of *pnp* and *secY* genes they enable analysis of haplotypes [9,18].

Unlike pear (*Pyrus*) and apple (*Malus*) species, where resistant genotypes to their respective phytoplasmas (‘*Ca*. P. pyri’ and ‘*Ca*. P. mali’) have been obtained [19,20], no resistance has been observed in *Prunus* species to ‘*Ca*. P. prunorum’. Nevertheless, high variability in symptom manifestation has been observed among species of this genus. In general, a high tolerance is observed in genotypes of European plum (*Prunus domestica*) and myrobalan (*Prunus cerasifera*), mild tolerance at damson plum (*Prunus insititia*) and high susceptibility at peach (*Prunus persica*) and apricot (*Prunus armeniaca*) [21,22,23]. Cherry (*Prunus avium*) and sour cherry (*Prunus cerasus*) are not showing any or only very weak ESFY symptoms and they also suppress the phytoplasma proliferation in their tissues [22,24]. In most of the studies, the response of *Prunus* species to ‘*Ca*. P. prunorum’ was based on the assessment of the symptoms and the phytoplasma presence confirmation by DAPI staining or end-point PCR detection. So far, there is no evidence that *Prunus* species showing only weak ESFY symptoms also have lower phytoplasma titer in their tissues. In case of lower phytoplasma titer, these species could be further used in breeding programs for the creation of highly tolerant and/or low phytoplasma titer rootstock–scion combinations.

The aim of this study was to characterize ‘*Ca*. P. prunorum’ genotypes by sequence analysis of the *aceF*, *pnp*, *imp* and *secY* genes in phytoplasma strains infecting *Prunus* species and its vector, *C. pruni*, collected in the Czech Republic. Furthermore, the two most common ‘*Ca*. P. prunorum’ haplotypes were then used for the inoculation of the most common apricot rootstocks to analyze their symptom manifestation, phytoplasma titer and dynamics of phytoplasma colonization during the growing season.

## 2. Materials and Methods

### 2.1. Plant and Insect Samples for ‘Ca. P. prunorum’ Molecular Characterization

**Figure 1:**
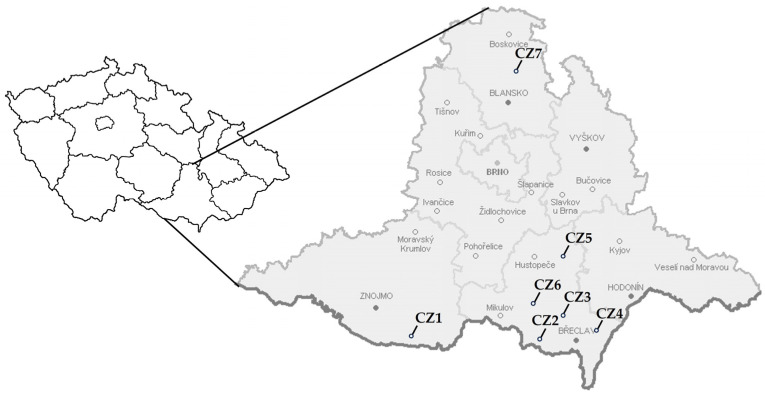
Sampling sites of *Prunus* trees and *C. pruni* individuals used for molecular characterization of ‘*Ca*. P. prunorum’.

For molecular characterization, 56 plant samples and 33 *C. pruni* individuals found positive for ‘*Ca*. P. prunorum’ were used (Tables S1 and S2). They were collected from 7 sampling sites (CZ1–CZ7) located in the region of the South Moravia (Figure 1), the main area of apricot production in the Czech Republic.

Plant samples were collected during the summer and autumn in 2006 or 2015 from ESFY symptomatic but also asymptomatic trees of four *Prunus* species (*P. armeniaca*, *P. persica* and *P. salicina* and *P. cerasifera*) grown in production or experimental orchards (Table 1). One plant sample consisted of 3 two-year-old shoots collected evenly from the tree canopy.

*C. pruni* individuals (emigrants and remigrants) were collected from 2003 to 2012 either in overwintering areas from *Picea* sp. trees (September to February) or in apricot growing areas from blackthorn (*Prunus spinosa*) and apricot trees (April to June) (Table 1). The collection was carried out by the beating tray method.

### 2.2. Plant Material for Rootstock Susceptibility Testing and Sampling

Three apricot rootstocks differing in their susceptibility to ‘*Ca*. P. prunorum’ were used in this trial. St Julien A (damson plum) was selected as the least susceptible rootstock, M-VA-1 (apricot selection) as susceptible and GF-305 (peach) as highly susceptible. The M-VA-1 and GF-305 rootstocks were propagated generatively, the St. Julien A rootstocks were a certified, i.e., phytoplasma and virus-free, vegetatively propagated plant material. All plants were grown under insect-proof net in pots (6 L volume) in a 3:4 mixture of topsoil and substrate (TS 3, Klasmann, Geeste, DE), irrigated by sprinklers and fertilized once a year with Yara Mila Complex (Yara, Oslo, NO) according to manufacturer’s instructions. Plants were inoculated in August 2013 by chip-budding separately with 2 different ‘*Ca*. P. prunorum’ isolates, one named as Poyer (A6-P2-I4-S2 haplotype, tree PR39—no symptoms, Table S1) and the second as Hargrand (A5-P2-I4-S2 haplotype, tree PR24—medium leafroll and chlorosis, Table S1). For each rootstock–phytoplasma isolate combination, 110 plants were used. As a negative control, 40 non-inoculated plants from each rootstock were used. Inoculated trees were maintained with growing rootstock and scion parts where only the rootstock part was evaluated.

Each year the leaf samples (4 leaves) were collected from annual shoots in June and October from 2015 to 2017 for quantification of ‘*Ca*. P. prunorum’ in tissues. Symptoms of ESFY were evaluated every year from 2015 to 2017 in the end of summer (early September). Leafroll and leaf chlorosis were analyzed separately according to a 4-point scale (1—no symptoms, 2—weak symptoms, 3—mild symptoms, 4—severe symptoms) (Figure S1). The individual values of leafroll and leaf chlorosis of each tree were then summed and expressed as disease index (DI).

### 2.3. DNA Extraction, Phytoplasma Detection and Quantification

Total plant DNA was extracted from phloem or leaf midrib tissue using a modified CTAB protocol according to Maixner et al. [25]. DNA pellets were resuspended in 100 µL of nuclease free water (Ambion, Austin, TX, USA). For the rootstock susceptibility test, approximately 0.3 g of leaf midrib tissue was weighed accurately to 1 mg on a precise scale Kern EG 620 3NM (Kern & Sohn, Balingen, DE) prior DNA extraction. Total insect DNA was extracted from individual psyllids, which were homogenized in Nuclei Lysis Solution buffer using micropestle homogenizer, using Wizard Genomic DNA Purification Kit (Promega, Madison, WI, USA) and eluted in 30 µL of deionized water.

For our molecular characterization study, the phytoplasma presence in samples was detected by nested PCR using P1/P7 primers [26,27] in the first and f01/r01 primers [28] in the second PCR as described in EPPO PM 7/62 [3]. The presence of targeted amplified DNA was confirmed by gel electrophoresis. The ‘*Ca*. P. prunorum’ specific detection was carried out using a real-time PCR protocol and the ESFY probe according to Nikolić et al. [29]. The Eco Real-Time PCR System (Illumina, San Diego, CA, USA) and EcoStudy Software 5.0 (Illumina, San Diego, CA, USA) was used for PCR amplification, fluorescence acquisition and identification of *ct* values. Each sample was tested in duplicates in all PCR assays.

In DNA samples from the rootstock susceptibility test, the real-time PCR protocol by Christensen et al. [30] was used for ‘*Ca*. P. prunorum’ detection and absolute quantification. A reaction of 10 μL total volume consisted of 0.3 μM of Forward primer, 0.9 μM of Reverse primer, 0.2 μM of TaqMan probe, 1× TaqMan Universal PCR Master Mix (Applied Biosystems, Foster City, MA, USA) and 1 μL of DNA. The thermal protocol consisted of polymerase activation for 10 min at 95 °C, followed by 40 cycles with 15 s at 95 °C, 60 s at 60 °C and plate read on FAM channel. To set up the standard curve, the artificial plasmid standard was commercially prepared by the ligation of ‘*Ca*. P. prunorum’ 16S rRNA Forward primer/Reverse primer amplicon [30] into pCR4-TOPO plasmid (Generi Biotech, Hradec Králové, Czech Republic). This standard was mixed with DNA from healthy GF-305 (peach) leaf midribs, extracted in the same way as mentioned above, at 10-fold serial dilution to obtain 10^7^ to 10^1^ copies·μL^−1^. The Eco Real-Time PCR System (Illumina, San Diego, CA, USA) and EcoStudy Software 5.0 (Illumina, San Diego, CA, USA) was used for fluorescence acquisition, determination of *ct* values and for computation of the absolute quantity of ‘*Ca*. P. prunorum’ that was expressed as number of phytoplasmal cells per gram of plant tissue (cells·g^−1^).

### 2.4. Molecular Characterization

In molecular characterization study, four marker genes (*aceF*, *pnp*, *imp* and *secY*) were used to characterize ‘*Ca*. P. prunorum’ in DNA samples. DNA fragments of each gene were amplified by nested PCR using the primers described in Danet et al. [9]. A reaction of 25 µL total volume consisted of 1 µL of DNA or 40 times diluted product of the 1st PCR used as an input for the 2nd PCR, 0.3 µM of each primer of a respective primer pair, 1.5 mM Mg^2+^ (Promega, Madison, WI, USA), 0.125 mM of each dNTP (Promega, Madison, WI, USA), 1 U GoTaq G2 polymerase (Promega, Madison, WI, USA), 1× Colorless GoTaq Flexi buffer (Promega, Madison, WI, USA) and nuclease free water (Ambion, Austin, TX, USA). PCR thermal conditions were kept the same as in Danet et al. [9]. All samples were analyzed in duplicates, after amplification the duplicate PCR reactions were merged and 10 µL of a product was checked by gel electrophoresis. In the case of a specific PCR product, the rest of the PCR reaction was purified using NucleoSpin Gel and PCR Clean-up (Macherey-Nagel, Düren, DE) and sequenced from both sides using Sanger sequencing in Eurofins Genomics (Ebersberg, DE). Obtained sequences were carefully checked for ambiguous bases, sequence pairs were joined and trimmed according to nested primers in CLC Main Workbench 23 (Qiagen, Hilden, DE). Sequences were compared with GenBank records using BLASTN [31].

The similar protocol was used for molecular characterization of ‘*Ca*. P. prunorum’ strains infecting *C. pruni* vectors. DNA fragments of each gene were amplified by nested PCR using the primers described in Danet et al. [9] in total volume of 25 µL using MyTaq Red DNA polymerase (Bioline, London, UK). Due to small volume of isolated DNA, the samples were not analyzed in duplicates, the presence of amplicon was confirmed by the electrophoretic separation of 2 µL of PCR reaction mix, in the presence of expected product, the amplicon was purified and subdued to bi-directional Sanger sequencing using BigDye Terminator v.3.1 Cycle Sequencing Kit and Genetic Analyser ABI Prism 3730 (both Applied Biosystems, Waltham, MA, USA) at the Sequencing Centre of Institute of Experimental Botany of the Czech Academy of Sciences, v. v. i. and Palacký University, Olomouc (CATRIN-UPOL). Obtained Sanger output sequences were carefully checked, trimmed and subsequently assembled into the final contigs, all using Geneious Prime assembler 2023.1.2 (Dotmatics, Boston, MA, USA).

Representative type sequences of identified genotypes per plant species were deposited in GenBank under Accession numbers PP176477–PP176488 for *aceF* gene, PP176489–PP176495 for *pnp* gene, PP176496–PP176508 for *imp* gene and PP176509–PP176515 for *secY* gene (Table S3).

### 2.5. Statistical Analysis

The haplotype network was constructed in PHYLOViZ 2.0 software (http://online.phyloviz.net, accessed on 2 December 2023) based on already published haplotypes and haplotypes obtained in this study. For haplotype network construction, only the samples where genotypes of all four marker genes were identified were used.

Data from phytoplasma quantification were analyzed using parametric tests. First the ANOVA in general linear models (GLM) was used for analysis of effects (*p* < 0.05) of independent variables or their interaction. Rootstock type, sampling year, ‘*Ca*. P. prunorum’ isolate and sampling season were considered as independent variables and for interaction analysis, the combinations of 2 independent variables were used. When a significant effect was observed, the differences between the variants were further analyzed by post hoc analysis with Tukey’s test (*p* < 0.05).

Disease index data were analyzed by non-parametric Kruskal–Wallis test (*p* < 0.05), where the differences between variants (combination of rootstock and phytoplasma genotype) were analyzed separately for each year. Parametric and non-parametric analyses were performed using Statistica 12 software (Tibco, Santa Clara, CA, USA).

## 3. Results

### 3.1. Diversity of Genotypes

Despite the presence of ‘*Ca*. P. prunorum’ in plant/insect samples and successful amplification of the analyzed genes, it was not possible to obtain good sequences in all cases and thus only those with clear sequences were used for diversity studies. In addition, the analyses of *aceF* and *imp* gene Sanger chromatograms allowed detection of double peaks corresponding to two-nucleotide assignments in polymorphic sites, and identification of mixed infection of more than one genotype in 8.5% and 10.6% of plant samples, respectively, all of them sampled in Lednice (CZ3, Table 2).

The BLASTN analysis confirmed the gene specificity of all sequences obtained, all of them were identical to each other within a particular genotype; the type sequences are listed in Table 2. They showed full identity with the specific *aceF*, *pnp*, *imp* and *secY* genotypes published previously [9,18] and are available in GenBank.

Of the four *aceF* genotypes detected in the Czech Republic, the A5 and A6 genotypes were the most common, occurring in in more than 78% of plant and 64% of insect samples (Table 2). These genotypes were found in almost all sampling sites. Lower frequencies of A3 and A8 genotypes were observed in samples from plants (2.1 and 10.6%, respectively) or insects (14.3 and 21.4%, respectively) and these genotypes were present in less than half of the sampling sites.

From the *pnp* gene, most of the isolates were P2 genotypes, which were present in 97.9% of the plant samples and 81.5% of insect samples collected from all sampling sites (Table 2). The P1 genotype was identified in only one (2.1%) isolate from plant and five (18.5%) isolates from insects.

Diversity of the *imp* gene (six genotypes) in Czech ‘*Ca*. P. prunorum’ isolates was the highest of all genes analyzed. The most frequent genotype was I4, which was detected in about half of plant (55.3%) and insect (46.4%) samples and it was present in six out of seven sampling sites (Table 2). Fifty percent of insects isolates from insects showed either I1 or I10 genotype, whereas isolates from plants had a lower representation of these genotypes (19.1%). I1 and I10 genotypes were identified in more than half of the sampling sites. Genotype I3 was the least represented genotype identified in both plant and insect samples. Genotypes I13 and I34 were detected exclusively in plant isolates sampled in Lednice and were present in more than 12% of the isolates.

More than 96% of plant samples and more than 78% of insect samples were infected with ‘*Ca*. P. prunorum’ S2 genotype and were collected in all sampling areas (Table 2). The S1 genotype was present only in one (1.9%) isolate from plants but in six (21.4%) isolates from insects in two sampling areas. The S3 genotype was again observed in only one isolate in plant from Lednice (CZ3).

### 3.2. Haplotype Diversity

In total, 11 different ‘*Ca*. P. prunorum’ haplotypes were identified in 55 samples (Table 3). Of these, A5-P2-I4-S2 and A6-P2-I4-S2 were the most frequent in both plant and insect samples. They were present in more than 62% of isolates from the plants and 50% from the insects collected at almost all sampling sites. The other haplotypes were not as widespread and were mostly identified in one or up to three sampling sites. Some of these haplotypes were unique to the source organism, where A3-P2-I1-S1 and A5-P2-I3-S2 haplotypes were detected exclusively in the insects and the A5-P2-I34-S2, A6-P2-I3-S2, A6-P2-I34-S2, A8-P1-I13-S3 haplotypes were detected exclusively in the plants. All these source specific haplotypes were only detected in one or two samples. The founder haplotype A3-P1-I1-S1 was present in both plant and insect samples, but at very low frequency (one and two samples, respectively).

**Figure 2:**
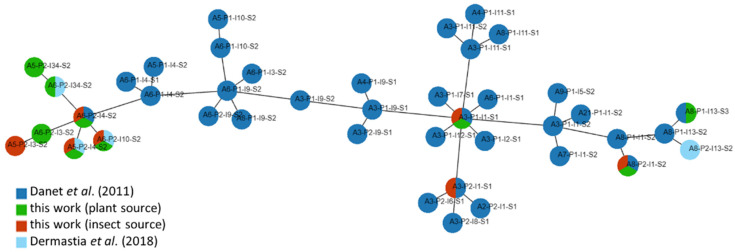
Network of known ‘*Ca*. P. prunorum’ haplotypes identified in this study and in the works of Danet et al. [9] and Dermastia et al. [18]. The network was created in the PHYLOViZ 2.0 software (http://online.phyloviz.net, accessed on 2 December 2023).

The haplotype network showed, that most of the haplotypes from the Czech Republic were closely related (Figure 2). These haplotypes consisted of A5 and A6 *aceF* genotypes and were present in more than 85% of isolates from plants and 75% of isolates from insects. The rest of the haplotypes, comprising A3 and A8 *aceF* genotypes, clustered in other parts of the network. New, unpublished haplotypes were observed in isolates from plants (A5-P2-I34-S2, A6-P2-I3-S2) and insects (A5-P2-I3-S2); however, each haplotype was present in only one isolate.

### 3.3. ‘Ca. P. prunorum’ Isolates in Asymptomatic Plants

**Figure 3:**
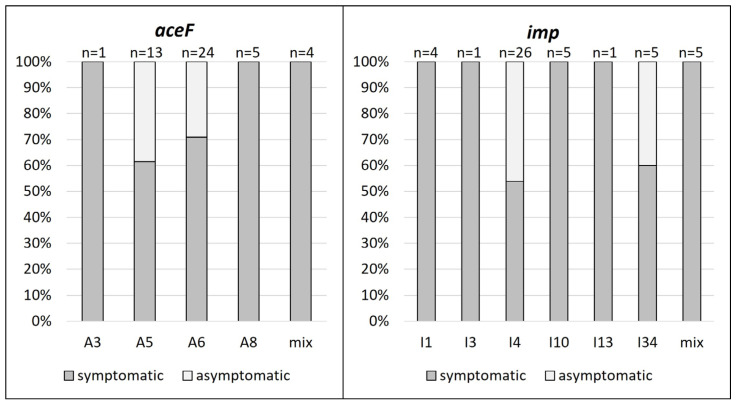
Representation of *aceF* and *imp* ‘*Ca*. P. prunorum’ genotypes in symptomatic and asymptomatic trees. Mix refers to mixed infections of multiple genotypes of respective gene.

Several genotypes were identified in asymptomatic trees; however, these genotypes were also present in symptomatic trees (Figure 3). For example, about 40% of isolates with A5 genotype and about 30% of isolates with A6 genotype were present in asymptomatic trees. For *imp* gene, a similar pattern was observed for I4 and I34 genotypes, where approximately 45% and 40% of isolates with respective genotypes were present in asymptomatic trees. The rest of *aceF* and *imp* genotypes were identified in symptomatic trees.

**Figure 4:**
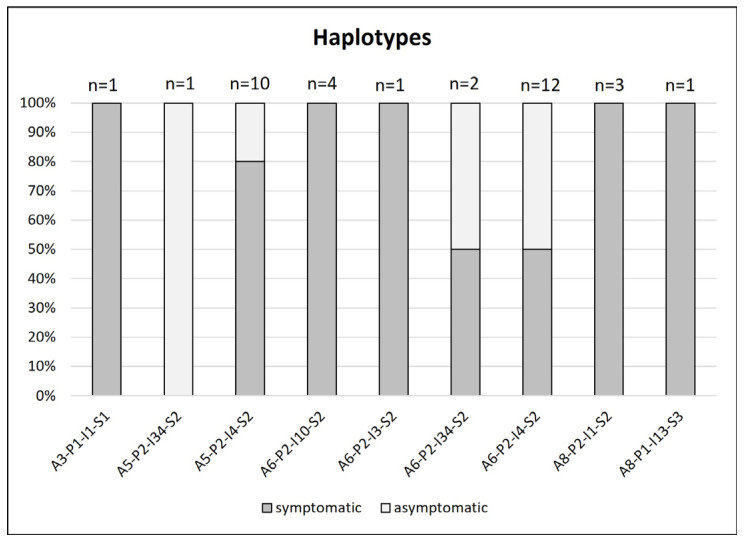
Representation of ‘*Ca*. P. prunorum’ haplotypes in symptomatic and asymptomatic trees.

Altogether, four haplotypes were identified in asymptomatic trees (Figure 4). Despite the presence of the A5-P2-I34-S2 and A6-P2-I34-S2 haplotypes in asymptomatic trees, their frequency was overall very low, being identified in only one and two trees, respectively. More reliable results were obtained for the A6-P2-I4-S2 haplotype, where six out of 12 trees in which it was identified were asymptomatic. A lower frequency of asymptomatic trees (two out of 10 trees) was observed for A5-P2-I4-S2 haplotype. All haplotypes from asymptomatic trees differed only in *aceF* and *imp* genotypes.

### 3.4. Rootstock Susceptibility Test

Out of 110 trees per rootstock–phytoplasma isolate combination (i.e., experimental variant) established in 2013, between 22 and 61 trees were used for evaluation in 2015 due to the decline of trees. The GF-305 rootstock variants consisted of 22 and 23 trees for the Poyer and Hargrand phytoplasma isolates, respectively, while the M-VA-1 and St. Julien A rootstock variants consisted of 41 to 61 trees (Figure S2). The negative control consisted of 40, 33 and 29 trees of St. Julien A, M-VA-1 and GF-305 rootstocks, respectively (Figure S2). In 2017, only between 22 and 42% of the number of trees in 2015 remained in the phytoplasma positive variants and between 30 and 45% in the negative variants tested. Nevertheless, at least five trees were always present in each variant.

A similar pattern of decrease in the number of trees was observed in both phytoplasma positive and negative variants of the respective rootstocks throughout the experimental period (2015–2017), which indicates that the tree decline was not mainly caused by the ‘*Ca*. P. prunorum’ presence in positive variants (Figure S2).

#### 3.4.1. Phytoplasma Titer

The phytoplasma titer in the rootstock tissues was significantly affected by the rootstock type (*p* ˂ 0.001), ‘*Ca*. P. prunorum’ isolate (*p* ˂ 0.001) and the sampling season (*p* ˂ 0.001), whereas only the sampling year had no influence on the phytoplasma titer (*p* = 0.559) (Table 4). In some cases, significant interactions were observed between particular factors (Table 4).

**Figure 5:**
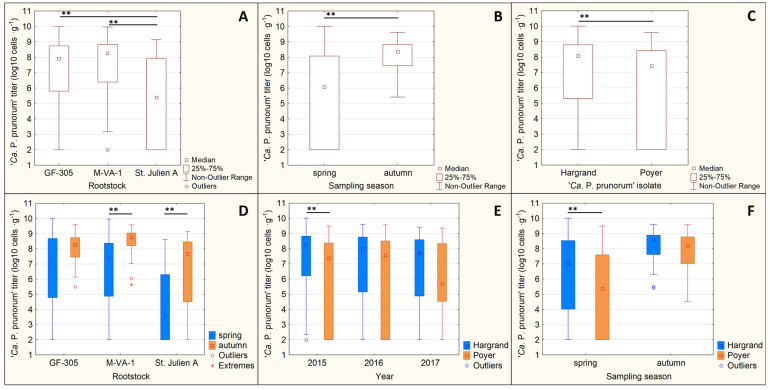
Results of ‘*Ca*. P. prunorum’ quantification in plant tissues. (**A**) the phytoplasma titer in tested rootstocks, (**B**) the phytoplasma titer in plant tissues in different sampling seasons, (**C**) the phytoplasma titer in plant tissues of Hargrand (A5-P2-I4-S2 haplotype) and Poyer (A6-P2-I4-S2 haplotype) ‘*Ca*. P. prunorum’ isolates, (**D**) the phytoplasma titer in tested rootstocks in different sampling seasons, (**E**) the phytoplasma titer of two ‘*Ca*. P. prunorum’ isolates in plant tissues each year separately and (**F**) shows pytoplasma titer of ‘*Ca*. P. prunorum’ isolates in plant tissues in different sampling seasons. Bars with asterisks indicate significant differences between variants based on ANOVA with subsequent post hoc Tukey test (** for *p*-value ˂ 0.01).

During the whole testing period, the St. Julien A rootstock contained significantly lower phytoplasma titer in analyzed tissues than M-VA-1 and GF-305 rootstocks (Figure 5A). There was no difference in phytoplasma titer between GF-305 and M-VA-1 rootstocks.

The results also show that ‘*Ca*. P. prunorum’ Hargrand isolate (A5-P2-I4-S2 haplotype) was multiplying in significantly higher numbers in plant tissues than the phytoplasma Poyer isolate (A6-P2-I4-S2 haplotype) (Figure 5C). However, this difference was observed only in the first test year (2015, Figure 5E) and only in spring sampling (Figure 5F).

Finally, ‘*Ca*. P. prunorum’ titer was significantly higher in the tissues in the autumn than in the spring period (Figure 5B). However, this effect was only observed at M-VA-1 and St. Julien A rootstocks (Figure 5D), whereas GF-305 showed constantly high phytoplasma titers in both sampling periods.

#### 3.4.2. Disease Index (DI)

**Figure 6:**
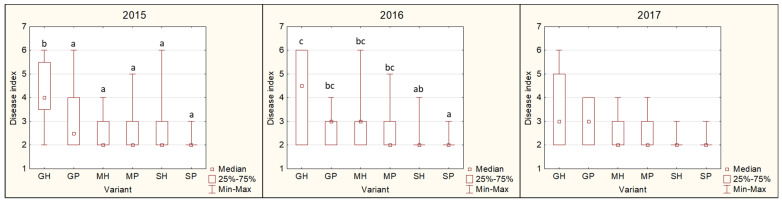
Disease index of tested rootstock–phytoplasma isolate combination in each testing year. GH is GF-305/Hargrand isolate combination, GP is GF-305/Poyer isolate combination, MH is M-VA-1/Hargrand isolate combination, MP is M-VA-1/Poyer isolate combination, SH is St. Julien A/Hargrand isolate combination and SP is St. Julien A/Poyer isolate combination. Letters above bars (a–c) indicate grouping of significantly different variants (*p* < 0.05) based on non-parametric Kruskal–Wallis test.

Throughout the test period (2015–2017), the GF-305 rootstock was showing higher DI values than the other rootstocks, regardless of the phytoplasma isolate (Figure 6). However, significantly higher DI values were observed only in 2015 and 2016 and not in all rootstock–phytoplasma isolate combinations. On the other hand, St. Julien A rootstock was showing very low or no symptoms, resulting in a low DI during the test period, where again, significant differences could only be observed in 2015 and 2016 for some variants. In 2017, no significant difference in DI was observed between the rootstock–phytoplasma isolate combinations.

## 4. Discussion

For better visualization, maps of ‘*Ca*. P. prunorum’ genotypes distribution in Europe and the Middle East were created from available data [9,12,13,18] (Figures S3–S6). These maps show that the distribution of some genotypes is area-wide while distribution of others is local.

Of the 12 *aceF* ‘*Ca*. P. prunorum’ genotypes identified so far [9], the A3, A5, A6 and A8 were detected in the Czech Republic. Two of these, the genotype A6, which is common in the Czech Republic together with the less common genotype A8, are widespread and were detected in almost all countries studied (Figure S3). Genotype A5, which is closely related to A6 [9] was reported only in the Central European countries (Austria, Czech Republic, Slovenia), and in Italy and Croatia. While the genotype A3 is the most common genotype in the Western Europe (Germany, France, Spain, Italy), it is rare in the Central European countries and was not detected in Greece, Turkey and Azerbaijan. There is a limited information on the frequency of *aceF* genotypes in the neighboring countries, Austria and Hungary, but A5 and A6 genotypes have been reported as the most frequent phytoplasma genotypes infecting apricots in these countries [12]. Nevertheless, the proportional distribution of Czech *aceF* genotypes is very similar to that in Slovenia, while very different from that of German *aceF* genotypes.

Only two *pnp* ‘*Ca*. P. prunorum’ genotypes have been identified up to date. Of these, the P1 genotype has been prevalent in all countries except in Slovenia and the Czech Republic, where the other genotype (P2) is the most frequent (Figure S4).

Out of 25 identified *imp* ‘*Ca*. P. prunorum’ genotypes [9,13,18], six genotypes (I1, I3, I4, I10, I13 a I34) were identified in the Czech Republic. The most frequent was the I4 genotype, which together with the I10 genotype, is mainly distributed in the Central Europe (Austria, Slovenia, Czech Republic), Italy, Croatia, and to lesser extent in France too (Figure S5). The I1 genotype is present in all countries surveyed except Spain and Turkey. Its frequency in the Czech Republic was lower than in the other countries, where it was mostly determined in around half or above half of the phytoplasma isolates. Genotypes I3 and I34 are only locally distributed and besides the Czech Republic, they were detected in Austria (I34 and I3), Germany (I3), Slovenia (I34) and France (I3 and I34). Finally, the genotype I13 has only scattered distribution (France, Italy, Croatia, Czech Republic) with a low proportional representation. The *imp* genotype composition and genotype frequencies in the Czech Republic are again more similar to those in Slovenia than in the other countries.

All three identified *secY* ‘*Ca*. P. prunorum’ genotypes were present in the Czech Republic. The most frequent genotype was S2, which is present in all countries analyzed (Figure S6). However, its proportional distribution in these countries is not the same. While in the western countries (Spain, France, Germany) its proportion is low, in the other countries (Italy, Czech Republic, Slovenia, Croatia, Azerbaijan) it is the most abundant genotype. In Spain, France and Germany, the most abundant genotype is the S1. Genotype S3 was reported in all countries, except Italy, Slovenia and Azerbaijan, but only in a few cases.

Based on observations only, the genotypic distribution of ‘*Ca*. P. prunorum’ seems to be not the same across Europe with different genotype composition and genotype frequencies for Central Europe compared to Western Europe. Several ways of spatial transmission of ‘*Ca*. P. prunorum’ have been described. While vector transmission is mostly local and not exceeding a radius of 50 km [13], the most probable explanation for long distance, interstate, transmission is the transmission by infected plant material [5,12,13]. Although current EU regulations prevent the spread of phytoplasma by propagating material, the spread of specific ‘*Ca*. P. prunorum’ genotypes on long distances could have happened, even before the regulations came into effect. For example, historically the European eco-geographical group of apricots has been divided into West European (i.e., Canino, Rouge du Rusillon, Luizet), East European (i.e., Hungarian best, Ananassa) and North European (Ukarinian ‘zherdeli’) subgroups [32]. These cultivars were mostly cultivated and transferred within geographically limited areas, the West European group in Western European and Mediterranean countries, the East European group in Central and Eastern European countries, and the North European group in Northeastern European countries. A similar historical pattern of cultivation and distribution was described for European plums [33]. Thus, in the past, the ‘*Ca*. P. prunorum’ genotypes could spread between the countries within the geographical area by the distribution of infected plant material, and the subsequent persistence of ‘*Ca*. P. prunorum’ genotypes in the area of cultivation could be maintained by the vector transmission. Although only a hypothesis, this is in line with previous suggestions of distribution of ‘*Ca*. P. prunorum’ between or within distant areas [13]. However, more information is needed to prove this hypothesis, especially from the other countries where genotyping has not yet been conducted.

In the haplotype network constructed from the Czech haplotypes and all available haplotypes [9,18], most of the Czech haplotypes clustered together, with the A6-P2-I4-S2 haplotype as the founder. This haplotype was first identified in the work of Danet et al. [9]. Within this cluster, we have identified three novel haplotypes in this study, with A5-P2-I34-S2 and A6-P2-I3-S2 detected in plant samples and A5-P2-I3-S2 in insect samples.

The A6-P2-I4-S2 haplotype has been associated in several cases with asymptomatic phenotype [9,18] and this was confirmed in this study too. In addition, in the same cluster with this haplotype the A5-P2-I34-S2, A5-P2-I4-S2 and A6-P2-I34-S2 haplotypes and the latter two with A6-P2-I10-S2 in Dermastia et al. [18] were detected in asymptomatic trees as well. However, the asymptomatic phenotype has been also associated with haplotypes clustering in other closer (A6-P2-I9-S2, A6-P1-I10-S2, A6-P1-I4-S2) or more distant (A3-P2-I1-S1) regions of the network [9,18]. A high degree of complexity is then introduced by the information that most of these haplotypes have also been identified in symptomatic trees. Thus, the identification of less virulent haplotypes based on the analysis of only four marker genes is not straightforward. Nevertheless, it seems that there is a higher probability of less virulent strains with A6 or A5 *aceF*, P2 *pnp*, I4, I10 and I34 *imp* and S2 *secY* genotypes, as these were the most frequent genotypes in asymptomatic trees.

In addition to the phytoplasma genotype, various factors could influence the expression of symptoms, such as the plant genotype [21,22,23,24,34], the ongoing recovery of the plant [35], the number of phytoplasma genotypes simultaneously infecting the plant [18,36], or the plant microbiome itself [37]. To test some of these assumptions we inoculated three types of apricot rootstocks differing in susceptibility to ‘*Ca*. P. prunorum’ with two different ‘*Ca*. P. prunorum’ haplotypes originating from asymptomatic (A6-P2-I4-S2 haplotype named Poyer) and symptomatic (A5-P2-I4-S2 haplotype named Hargrand) apricot trees. Both haplotypes were the most frequent haplotypes in the Czech Republic. The results then proved, that the genotype of the phytoplasma influences its ability to multiply in plant tissues and the manifestation of symptoms as well. However, the effect was not significant throughout the whole observation period. The significance in phytoplasma titer and DI was proved only in the first year, while in the following years, although lower titer and DI was observed in Poyer-infected trees, the differences were not significant.

Rootstock type had higher influence on phytoplasma titer and symptoms manifestation than the phytoplasma genotype itself. Here, the St. Julien A rootstock (damson plum) significantly altered the phytoplasma proliferation and had the lowest DI, although not always significantly over the whole test period. The phytoplasma titer in M-VA-1 (apricot) and GF-305 (peach) plants was not different, while the DI was higher in GF-305, but again not significantly over the whole period. The observed tolerance of the plum rootstocks and the susceptibility of the apricot and peach rootstocks are consistent with the previous results [21,22,38,39,40]. These observations show that specific *Prunus* genotypes can result in low symptom manifestation while harboring low phytoplasma titers. Since variations in the susceptibility of apricot cultivars have been reported [34,41], a similar effect on the reduction in phytoplasma titer could be expected for tolerant varieties showing weak or no symptoms. The combinations of tolerant rootstock–cultivar could then create vital apricot trees with low phytoplasma titer and weak or no symptoms.

Finally, the phytoplasma titer was increasing in the plant tissues of apricot (M-VA-1), damson plum (St. Julien A) and in peach (GF-305), although not always significantly, throughout the vegetation period. This is in accordance with the previously observed increase in phytoplasma titers in apricots, peaches and Asian plums (*P. salicina*) during the vegetation period (July–September) [23]. In the same study, the phytoplasma titer was not increasing in *Prunus tomentosa* tissues, where the titer remained at low concentration during the whole vegetation period while the plants were showing only mild symptoms.

## 5. Conclusions

This is the first study focused on genotype identification of ‘*Ca*. P. prunorum’ isolates from plant and insect samples in the Czech Republic. The highest genotype diversity was observed for the *imp* (six genotypes) and *aceF* (four genotypes) genes and lower for the *secY* (three genotypes) and *pnp* (two genotypes) genes. The results also showed that the most abundant genotypes in both plant and vector samples were the same (A6, I4, S2, P2), while some of the less abundant genotypes were specific to plants (I13, I34, S3). Similarly, the most abundant haplotypes in both host organisms were the same haplotypes (A5-P2-I4-S2 and A6-P2-I4-S2), while some low abundant haplotypes were specific to plant (A5-P2-I34-S2, A6-P2-I3-S2, A6-P2-I34-S2, A8-P1-I13-S3) or insects (A3-P2-I1-S1, A5-P2-I3-S2). The distribution of genotypes and haplotypes in the Czech Republic is more similar to other countries of Central Europe than to the countries of Western Europe, which could be the result of the historical distribution of eco-geographical groups of *Prunus* species. Most of the Czech haplotypes were closely related as they clustered with the founder haplotype A6-P2-I4-S2 in the haplotype network. In asymptomatic trees, the same phytoplasma genotypes (i.e., A5, A6, I34, I4, S2, P2) were detected as in previous works. However, these genotypes were also present in symptomatic trees. The susceptibility test, in which two isolates representing the two most common Czech haplotypes were used to inoculate three types of apricot rootstocks, showed that the St. Julien A had decreased symptoms but also decreased phytoplasma titer in its tissue compared to the other rootstocks (M-VA-1 and GF305) in the whole test period. The analysis of the influence of the phytoplasma genotype was significant, but not over the whole test period. The results also showed that the phytoplasma titer in the plant tissues was increasing during the vegetation period.

## Figures and Tables

**Table 1:** Sampling sites and the number of samples from plant and insect species.

Sampling Site	Area	Host Species	Plant Samples	*C. pruni*	
				Emigrants	Remigrants
CZ1	Slup	*P. armeniaca*, *P. persica*	3	-	-
CZ 2	Valtice	*P. armeniaca*, *P. persica*	11	-	-
CZ 3	Lednice	*P. armeniaca*, *P. persica*, *P. salicina*, *P. cerasifera*	38	8	7
CZ 4	Hrušky	*P. armeniaca*	1	-	-
CZ 5	Kobylí	*P. armeniaca*	3	-	-
CZ 6	Bulhary	*P. spinosa*	-	-	3
CZ 7	Kalečník	*Picea* sp.	-	5	10
Total			56	13	20

**Table 2:** Diversity of ‘*Ca*. P. prunorum’ genotypes in the Czech Republic and their frequency rate in tested samples.

Gene	Genotype ^1^	GenBank Acc. No. of Type Sequences Obtained in This Study	Plant Source (No. of Samples/%)	Insect Source (No. of Samples/%)	Sampling Site (CZ)
*aceF*	A3	PP176477, PP176481	1/2.1	4/14.3	5, 7
	A5	PP176478, PP176482	13/27.7	9/32.1	1, 2, 3, 5, 6, 7
	A6	PP176479, PP176483, PP176486, PP176488	24/51.1	9/32.1	2, 3, 5, 6, 7
	A8	PP176480, PP176484, PP176485, PP176487	5/10.6	6/21.4	1, 3, 7
	mix ^2^		4/8.5	-	3
	Total		47/100	28/100	
*pnp*	P1	PP176489, PP176491	1/2.1	5/18.5	3, 5, 7
	P2	PP176490, PP176492–PP176495	47/97.9	22/81.5	1, 2, 3, 4, 5, 6, 7
	Total		48/100	27/100	
*imp*	I1	PP176496, PP176500, PP176506	4/8.5	10/35.7	2, 3, 5, 7
	I3	PP176497, PP176501	1/2.1	1/3.6	3, 6
	I4	PP176498, PP176502, PP176507, PP176508	26/55.3	13/46.4	1, 2, 3, 5, 6, 7
	I10	PP176499, PP176503	5/10.6	4/14.3	3, 4, 6, 7
	I13	PP176505	1/2.1	-	3
	I34	PP176504	5/10.6	-	3
	mix ^2^		5/10.6	-	3
	Total		47/100	28/100	
*secY*	S1	PP176509, PP176511	1/1.9	6/21.4	5, 7
	S2	PP176510, PP176512, PP176514, PP176515	52/96.3	22/78.6	1, 2, 3, 4, 5, 6, 7
	S3	PP176513	1/1.9	-	3
	Total		54/100	28/100	

^1^ Genotypes annotated according to Danet et al. [9] and Dermastia et al. [18] ^2^ Mixed infection of at least two genotypes in one sample.

**Table 3:** Haplotype diversity of ‘*Ca*. P. prunorum’ isolates in the Czech Republic and their frequency rate in tested samples.

Haplotype	Plant Source (No. of Samples/%)	Insect Source (No. of Samples/%)	Sampling Site (CZ)
A3-P1-I1-S1	1/2.9	2/10	5, 7
A3-P2-I1-S1	-	1/5	7
A5-P2-I3-S2	-	1/5	6
A5-P2-I4-S2	10/28.6	6/30	1, 2, 3, 5, 6, 7
A5-P2-I34-S2	1/2.9	-	3
A6-P2-I3-S2	1/2.9	-	3
A6-P2-I4-S2	12/34.3	4/20	2, 3, 5, 7
A6-P2-I10-S2	4/11.4	3/15	3, 6, 7
A6-P2-I34-S2	2/5.7	-	3
A8-P1-I13-S3	1/2.9	-	3
A8-P2-I1-S2	3/8.6	3/15	1, 3, 7
Total	35/100	20/100	

**Table 4:** Analysis of variance (ANOVA) of single factors and interactions between factors in the analysis of phytoplasma titer in rootstock tissues. Factors and interactions with significant effects on phytoplasma titer are marked with * (*p*-value ˂ 0.05) and ** (*p*-value ˂ 0.01).

ANOVA	Factor	F-Value	*p*-Value
Single factor	rootstock type	61.022	˂0.001 **
	‘*Ca*. P. prunorum’ isolate	29.375	˂0.001 **
	sampling year	0.581	0.559
	sampling season	99.36	˂0.001 **
Interaction between factors	rootstock type × ‘*Ca*. P. prunorum’ isolate	1.713	0.181
	rootstock type × sampling year	0.884	0.473
	rootstock type × sampling season	3.998	0.019 *
	‘*Ca*. P. prunorum’ isolate × sampling year	4.634	0.010 *
	‘*Ca*. P. prunorum’ isolate × sampling season	4.663	0.031 *
	sampling year × sampling season	2.195	0.112

## Data Availability

The DNA sequences were deposited in the public database GenBank under Accession numbers PP176477–PP176515.

## Supplementary Materials

The following supporting information can be downloaded at: https://www.mdpi.com/article/10.3390/microorganisms12020399/s1, Figure S1: Scale for ESFY symptom evaluation of (A) leaf chlorosis and (B) leafroll: 1—no symptoms, 2—weak symptoms, 3—mild symptoms, 4—severe symptoms; Figure S2: Number of plants during the testing period (2015–2017) used for symptom and phytoplasma titer analysis in rootstock susceptibility test; Figure S3: ‘*Ca*. P. prunorum’ *aceF* genotype distribution across the Europe and Middle East countries; Figure S4: ‘*Ca*. P. prunorum’ *pnp* genotype distribution across the Europe and Middle East countries; Figure S5: ‘*Ca*. P. prunorum’ *imp* genotype distribution across the Europe and Middle East countries; Figure S6: ‘*Ca*. P. prunorum’ *secY* genotype distribution across the Europe and Middle East countries; Table S1: List of ‘*Ca*. P. prunorum’ isolates identified in plant samples; Table S2: List of ‘*Ca*. P. prunorum’ isolates identified in insect samples; Table S3: List of GenBank Accession numbers obtained in this study.
