# Supplementary figures and images for: Molecular Characterization of ‘Candidatus Phytoplasma prunorum’ in the Czech Republic and Susceptibility of Apricot Rootstocks to the Two Most Abundant Haplotypes
Source: Microorganisms. 2024 Feb 17;12(2):399. doi: 10.3390/microorganisms12020399 (PMC10893538; doi:10.3390/microorganisms12020399)

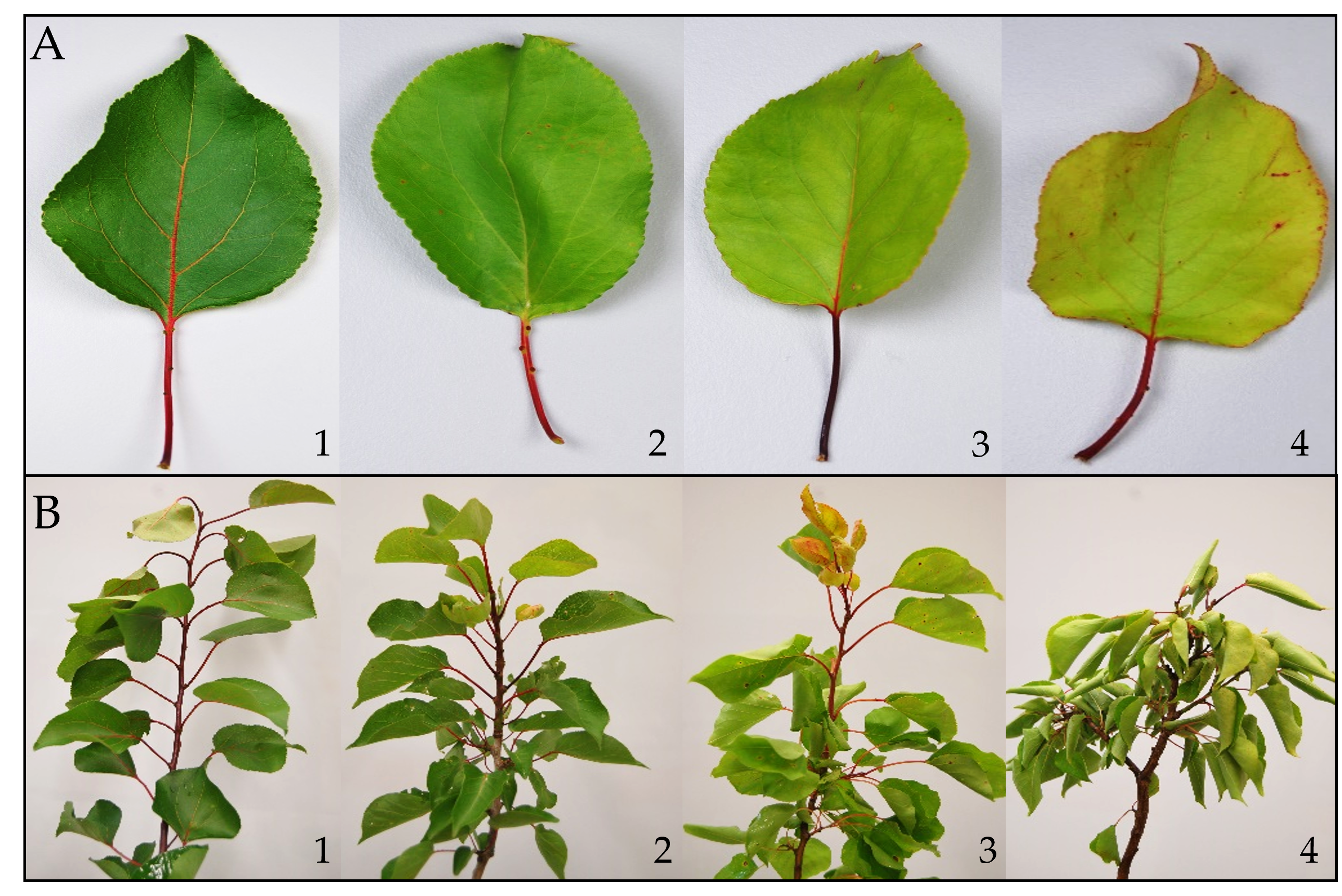

Supplement: Supplementary file 1 [file microorganisms-12-00399-s001.zip › Figure S1.tif]

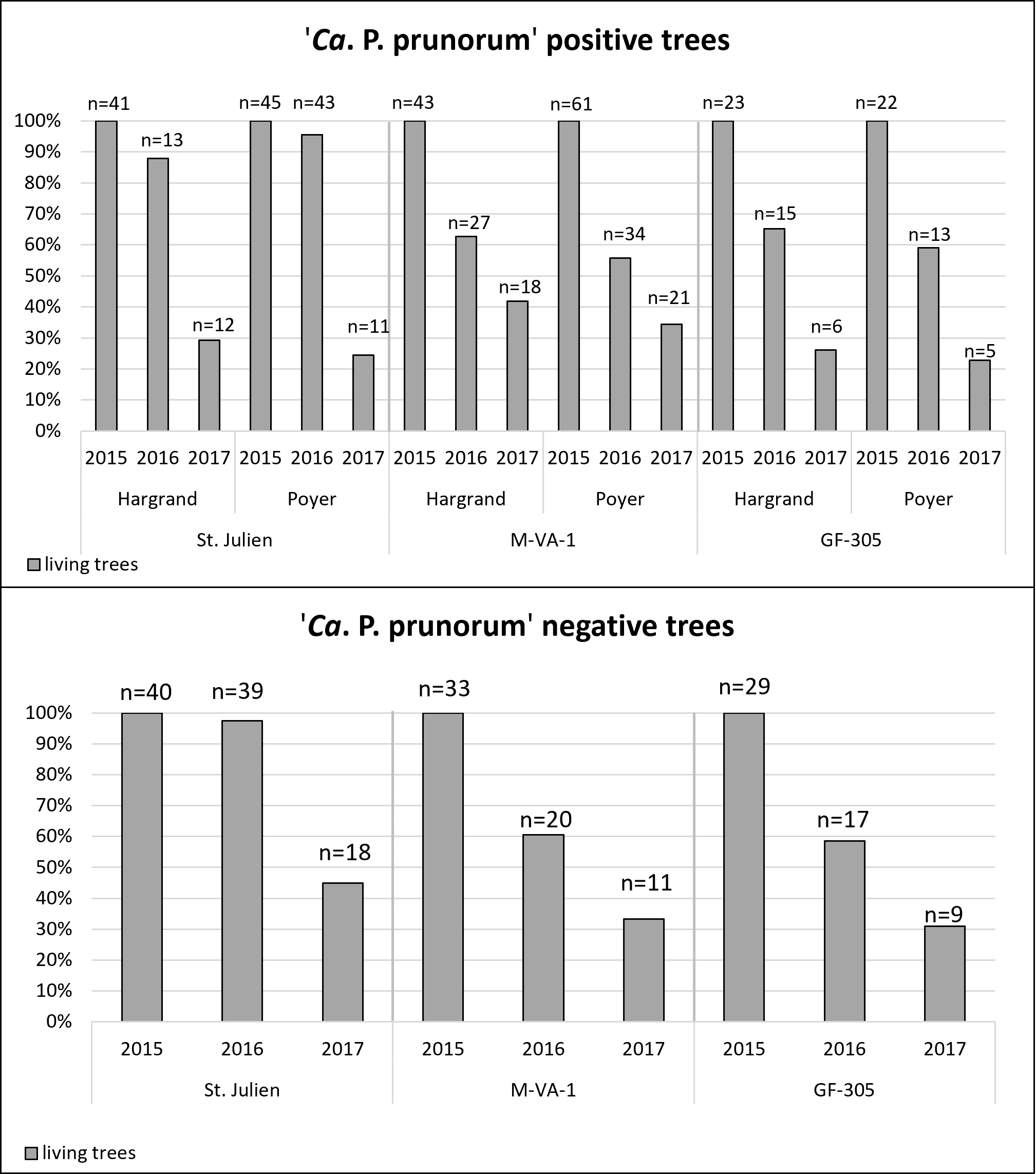

Supplement: Supplementary file 1 [file microorganisms-12-00399-s001.zip › Figure S2.tif]

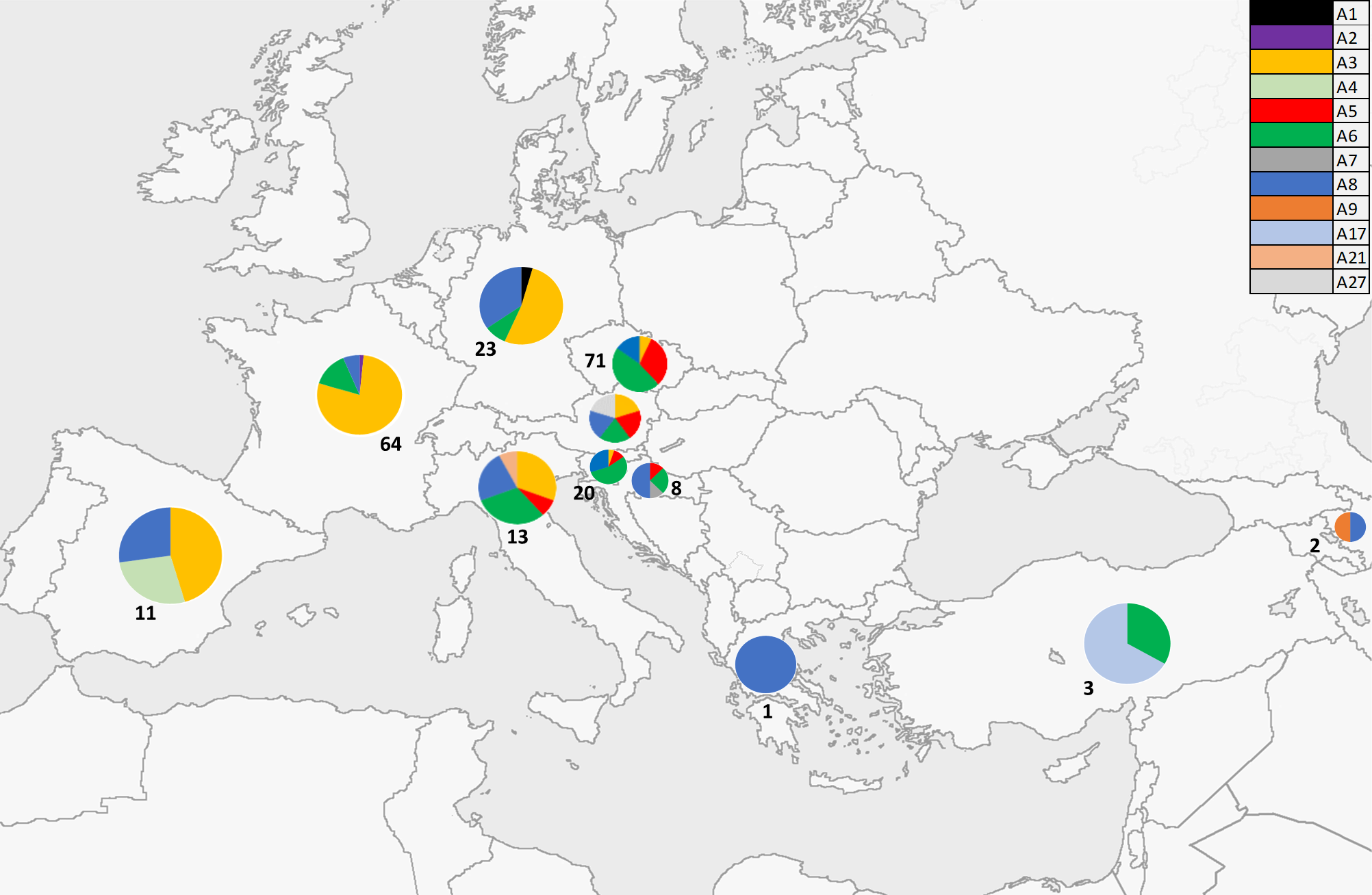

Supplement: Supplementary file 1 [file microorganisms-12-00399-s001.zip › Figure S3.tif]

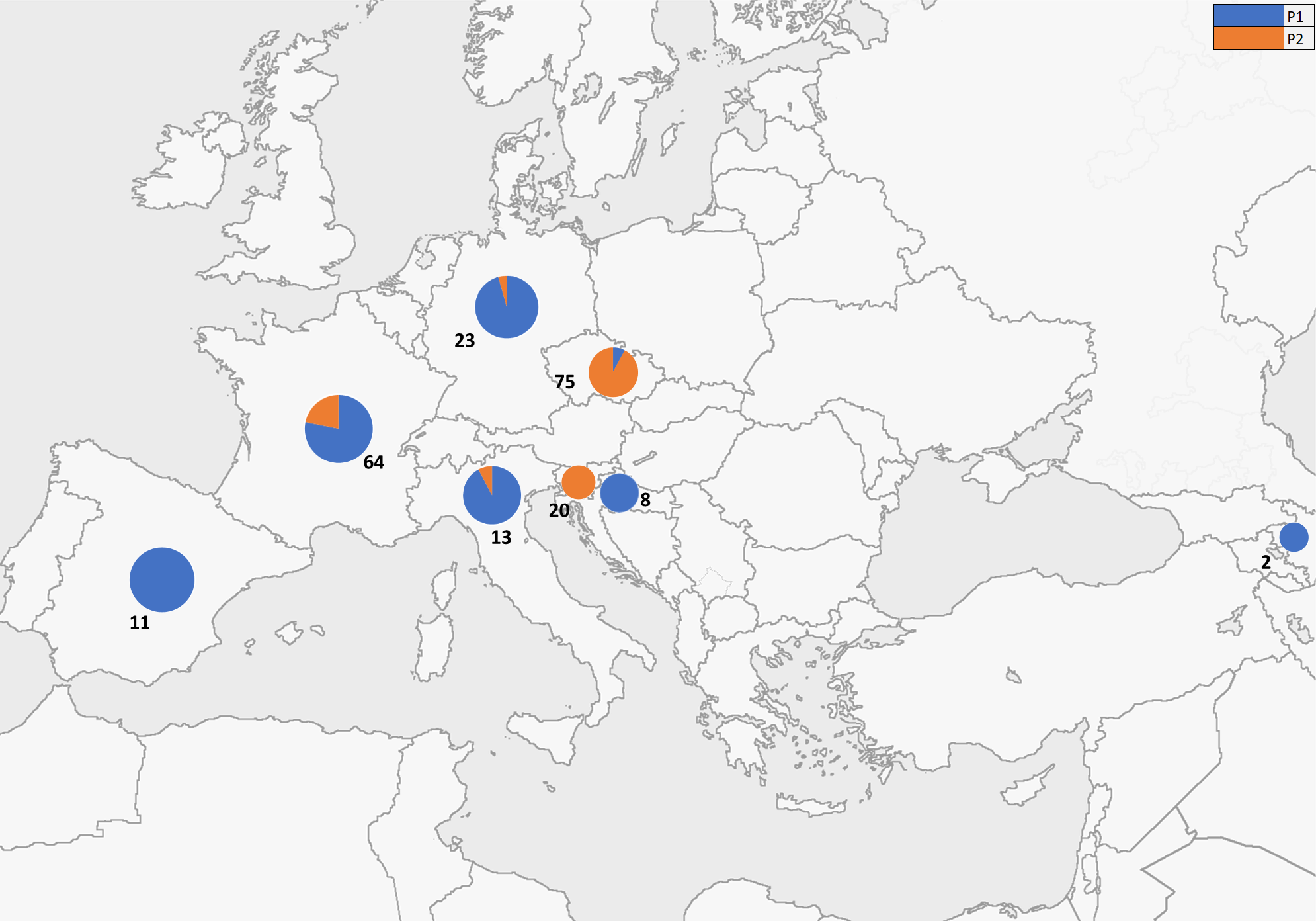

Supplement: Supplementary file 1 [file microorganisms-12-00399-s001.zip › Figure S4.tif]

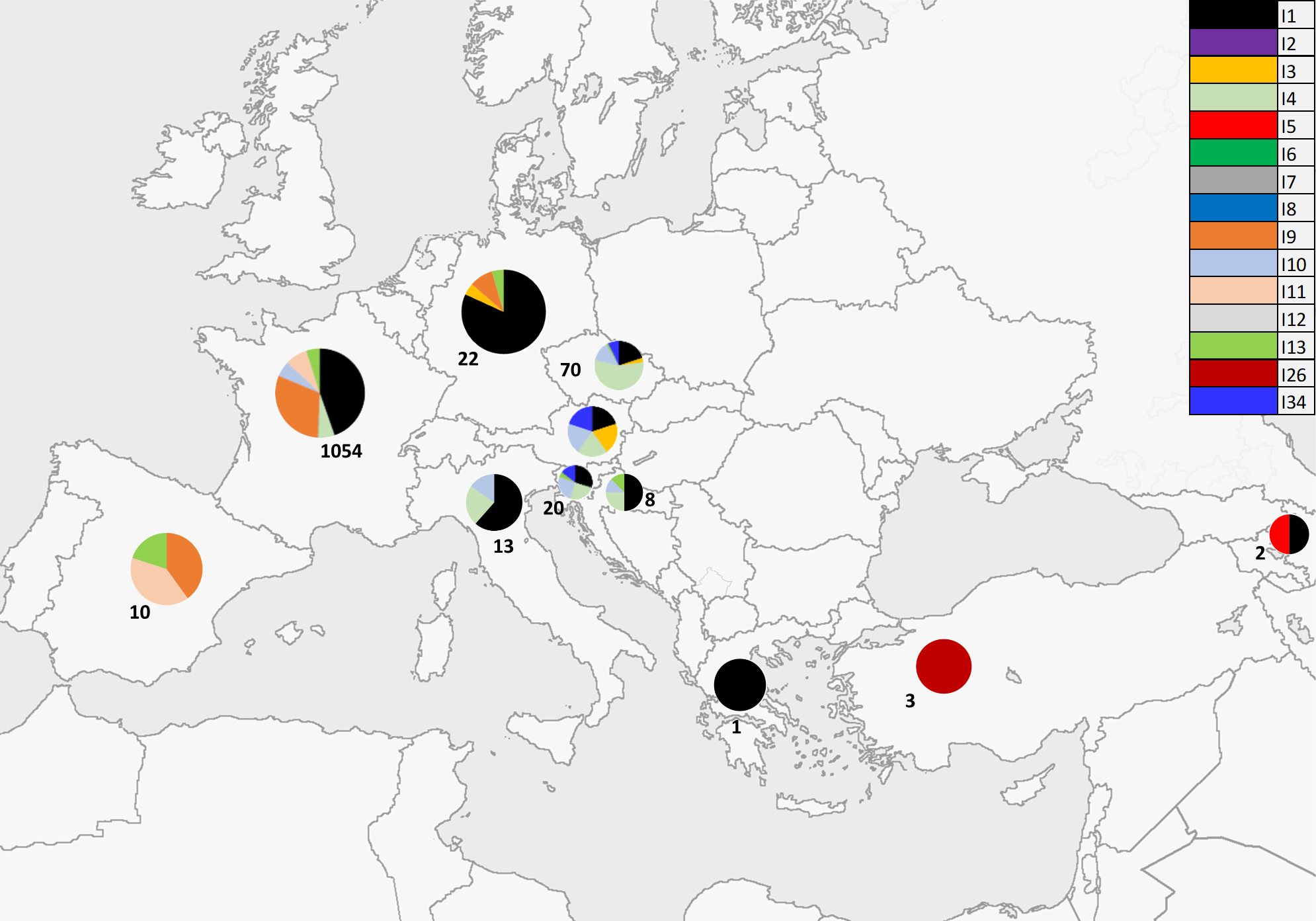

Supplement: Supplementary file 1 [file microorganisms-12-00399-s001.zip › Figure S5.tif]

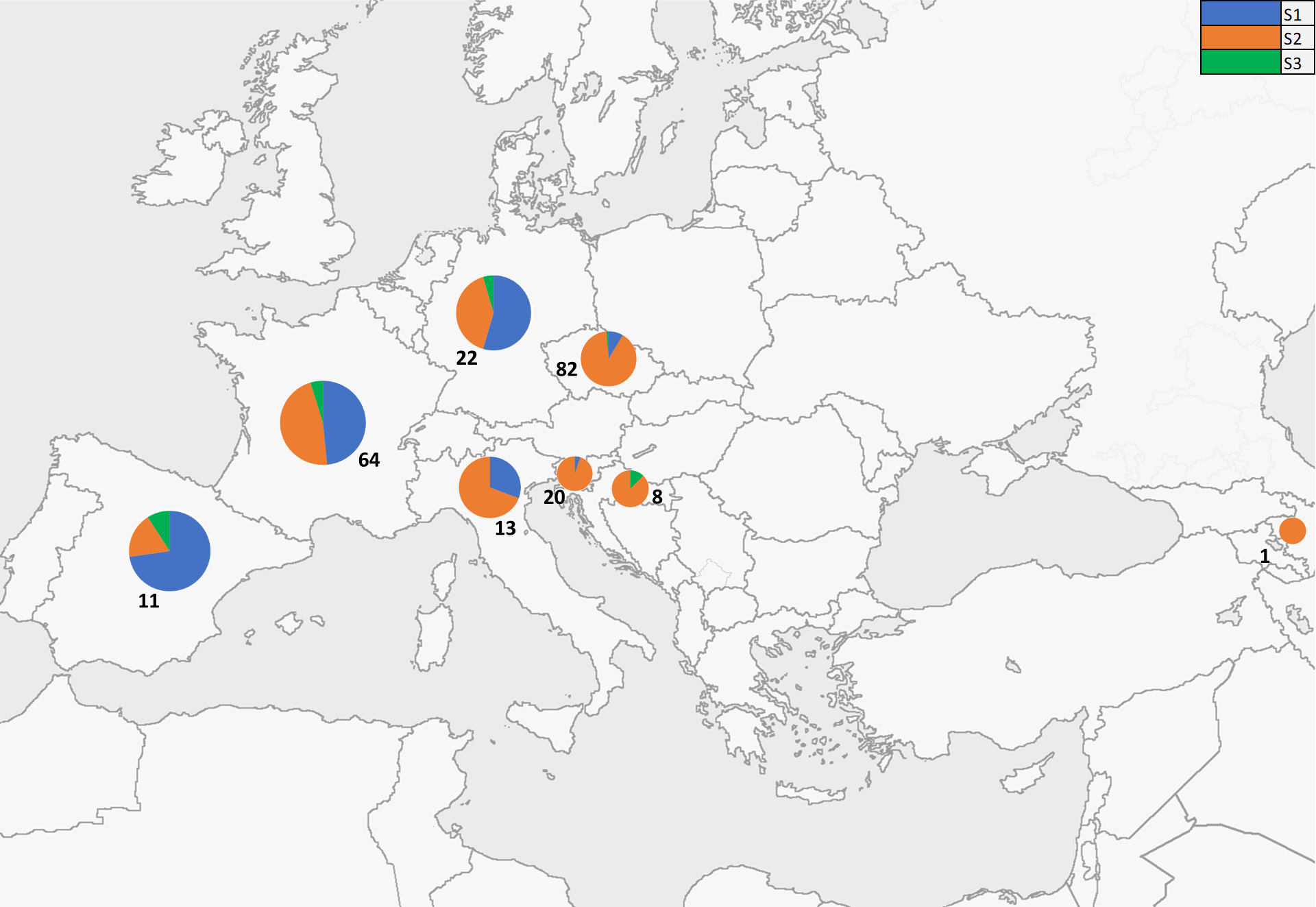

Supplement: Supplementary file 1 [file microorganisms-12-00399-s001.zip › Figure S6.tif]
